# Maternal Prenatal Infection and Anxiety Predict Neurodevelopmental Outcomes in Middle Childhood

**DOI:** 10.1037/abn0000746

**Published:** 2022-03-03

**Authors:** Thomas G. O’Connor, Allison Avrich Ciesla, Ana Vallejo Sefair, Loralei L. Thornburg, Alan S. Brown, Vivette Glover, Kieran J. O’Donnell

**Affiliations:** 1 Department of Psychiatry, University of Rochester; 2 Department of Psychology, University of Rochester; 3 Department of Neuroscience, University of Rochester; 4 Department of Obstetrics and Gynecology, University of Rochester; 5 Wynne Center for Family Research, University of Rochester; 6 Department of Public Health Sciences, University of Rochester; 7 New York State Psychiatric Institute, Columbia University Medical Center; 8 Institute of Reproductive and Developmental Biology, Imperial College London; 9 Yale Child Study Center, Yale University; 10 Department of Obstetrics, Gynecology and Reproductive Sciences, Yale University

**Keywords:** developmental programming, prenatal anxiety, prenatal infection, immune activation, ALSPAC

## Abstract

Prenatal maternal infection and anxiety have been linked, in separate lines of study, with child neurodevelopment. We extend and integrate these lines of study in a large prospective longitudinal cohort study of child neurodevelopment. Data are based on the Avon Longitudinal Study of Parents and Children (ALSPAC) cohort; prenatal maternal anxiety was assessed from self-report questionnaire; prenatal infection was derived from reports of several conditions in pregnancy (*n* = 7,042). Child neurodevelopment at approximately 8 years of age was assessed by in-person testing, reports of social and communication problems associated with autism, and psychiatric evaluation. Covariates included psychosocial, demographic, and perinatal/obstetric risks. Prenatal infection was associated with increased likelihood of co-occurring prenatal risk, including anxiety. Regression analyses indicated that both prenatal infection and prenatal anxiety predicted child social and communication problems; the predictions were largely independent of each other. Comparable effects were also found for the prediction of symptoms of attention problems and anxiety symptoms. These results provide the first evidence for the independent effects of prenatal infection and anxiety on a broad set of neurodevelopmental and behavioral and emotional symptoms in children, suggesting the involvement of multiple mechanisms in the prenatal programming of child neurodevelopment. The results further underscore the importance of promoting prenatal physical and mental health for child health outcomes.

## General Scientific Summary

There is considerable interest in understanding how and if common prenatal exposures may shape child neurodevelopment; results of these studies provide essential insight into developmental mechanisms and treatment/prevention strategies. The current study, based on a large community sample followed since pregnancy, provides the first evidence that two common prenatal exposures, maternal anxiety and infection, independently predict early-emerging signs of social and cognitive problems in children.

Prenatal influences on child neurodevelopment and psychiatric disorder are attracting considerable scientific and public health attention because of what the findings imply about mechanisms of development and the role of timing in (preventive) interventions (Arango et al., 2018; Marín, 2016; White, 2019). The current study advances research on two prenatal influences that have garnered particular interest. The first of these is prenatal maternal anxiety (some studies also include other measures of psychological distress, such as prenatal stress), which has been reliably associated with child neurodevelopment and psychiatric symptoms in large-scale longitudinal studies in several countries (King et al., 2009; O’Donnell et al., 2014; Robinson et al., 2008). The second is prenatal infection, which has been reliably associated with clinical conditions such as autism and schizophrenia (Allswede et al., 2020; Brown et al., 2004; Zerbo et al., 2013). The current study provides a valuable extension and integration of this work in the Avon Longitudinal Study of Parents and Children (ALSPAC) study, a large prospective longitudinal birth cohort study of a community sample that has followed mothers and children since the prenatal period.

Maternal prenatal distress, in the form of elevated anxiety or depressive symptoms or stress, has been reliably associated with child neurodevelopment and psychiatric symptoms for two decades (O'Connor et al., 2002). Much of this work derives from the conceptual model of developmental programming or Developmental Origins of Health and Disease (DOHaD; Barker, 1993; Gluckman & Hanson, 2005; Jones et al., 2011). In brief, the model proposes that early exposures instigate an adaptive response in the fetus with carry-forward effects to shape long-term health and development. Early studies of this model targeted prenatal nutritional environment and cardio-metabolic health outcomes (Lumey et al., 2011; Marques et al., 2013; Thurner et al., 2013), and has since been expanded to neurodevelopment outcomes (Bale et al., 2010; O’Donnell et al., 2009; Van den Bergh et al., 2020). The most widely-reported findings concern neurocognitive abilities (Buss et al., 2011; Laplante et al., 2008) and emotional and behavioral symptoms (O’Donnell et al., 2014; Walder et al., 2014). Experimental animal data suggest that the link between prenatally induced stress on offspring neurodevelopment may be explained by alteration in the Hypothalamic Pituitary Adrenal (HPA) axis and glucocorticoid exposure (Emack & Matthews, 2011; Schneider et al., 1992), although support for this mechanism in human studies is limited (e.g., O’Donnell et al., 2012).

A separate line of investigation based on the Maternal Immune Activation (MIA) model implicates a role for the prenatal maternal immune system on offspring neurodevelopment. Animal studies demonstrate that experimentally inducing immune activation or inflammation during pregnancy is associated with altered neurogenesis and reduced offspring brain volume (Bland et al., 2010; Malkova et al., 2012; Monje et al., 2003); nonhuman primate studies confirm that infecting pregnant rhesus monkeys with influenza reduced gray and white matter in the offspring (Short et al., 2010). Importantly, in the case of influenza, evidence suggests that the effect on the offspring derives from the maternal immune response—such as an elevated level of proinflammatory cytokines, which may cross the placenta and directly inhibit neurogenesis (e.g., Monje et al., 2003)—rather than the virus itself. That is an important observation because maternal immune activation may be associated with a wide range of exposures beyond infection, such as psychological distress, obesity, and other exposures such as pollution (e.g., Blackmore et al., 2011; Christian et al., 2009). Human data supporting the MIA model for offspring neurodevelopment include an influential set of studies showing that prenatal infection sizably increases the risk of psychotic disorders in offspring (Allswede et al., 2020; Brown et al., 2004; Lee, Cherkerzian, et al., 2020); other studies suggest that prenatal maternal febrile illness and infection are associated with autism and developmental delay (Zerbo et al., 2013, 2015). Consistent with the MIA hypothesis is the observation that other inflammatory conditions, such as prepregnancy obesity, are associated with child neurodevelopmental problems, including ADHD and autism (Sanchez et al., 2018); furthermore, child neurodevelopmental disorders may be more common if the mother has an autoimmune condition (Patel et al., 2020). More direct evidence for inflammation as potential causal agent is suggested by studies linking levels of cytokines and other inflammatory markers with neurodevelopmental conditions and cognitive outcomes (Brown et al., 2014; Ghassabian et al., 2018).

Despite their shared focus on the prenatal exposure period and on offspring neurodevelopment, research on prenatal maternal anxiety, on one hand, and infection or inflammation, on the other, has proceeded independently. In this study, we conjoin and contrast these competing hypotheses for three reasons. One reason is to examine potential confounding between these exposures. Overlap between these exposures would mean that causal inferences may be mis-specified. Infection in pregnancy is common—64% according to one national sample (Collier et al., 2009)—and so presents a potential confound in studies of other prenatal risk exposures. Reciprocally, mood disorders in pregnancy are common (Ross & McLean, 2006; Woody et al., 2017) and may confound the impact of alternative prenatal risk exposures for child health outcomes.

The second is to expand each line of study. In the case of research on prenatal infection and the MIA model, human studies have typically focused on limited clinical phenotypes, namely, schizophrenia and autism, with much less attention to less severe neurodevelopmental phenotypes and individual differences within the normal range (Gustavson et al., 2019; Lee, Papandonatos, et al., 2020). Studies of affective symptoms and disruptive behavior are particularly rare; that is a significant limitation insofar as understanding the nature of the risk mechanisms is informed by studying the range of outcomes to which a risk exposure is, and is not, predictive. Accordingly, in the current study we examine the phenotypes to include symptoms of affective and disruptive behavioral disorders. Studies of prenatal anxiety, in contrast, focus on common neurocognitive, behavioral, and emotional disturbances; we extend this line of study to consider autistic traits.

Third, we examine both prenatal infection and prenatal anxiety in order to consider possible mediating mechanisms. Neurodevelopmental effects on offspring associated with prenatal maternal anxiety or infection are proposed to derive from different focal mechanisms. For prenatal anxiety the leading mediating mechanism is presumed to be stress-related physiology and particularly the hypothalamic-pituitary-adrenal (HPA) axis (Glover et al., 2010; Kapoor et al., 2006); for maternal infection, the presumed mechanism is the maternal inflammatory immune response (Zerbo et al., 2014). These biological processes—HPA activity and inflammation—are not independent, however; moreover, an inflammatory pathway could plausibly explain the effects of prenatal maternal distress and an HPA-mediated mechanism could plausibly explain the effects of prenatal infection. Regardless of the degree of shared or distinct underlying mechanisms, research is needed to examine if prenatal anxiety and prenatal infection represent overlapping or distinct risks for child neurodevelopment; that is a novel aim of the current study.

## Method

### Sample

Data for this study were obtained as part of the Avon Longitudinal study of Parents and Children (ALSPAC), an ongoing population-based study designed to investigate the effects of a wide range of influences on the health and development of children (Boyd et al., 2013; Fraser et al., 2013). The study website contains details of all the data that is available through a fully searchable data dictionary and variable search tool and reference the following web page: http://www.bristol.ac.uk/alspac/researchers/our-data/. Pregnant women resident in Avon, U.K. with expected dates of delivery 1st April 1991 to 31st December 1992 were invited to take part in the study. The initial number of pregnancies enrolled is 14,541 (for these at least one questionnaire has been returned or a “Children in Focus” clinic had been attended). Of these initial pregnancies, there was a total of 14,676 fetuses, resulting in 14,062 live births and 13,988 children who were alive at 1 year of age. When the oldest children were approximately 7 years of age, an attempt was made to bolster the initial sample with eligible cases who had failed to join the study originally. As a result, when considering variables collected from the age of 7 onward (and potentially abstracted from obstetric notes) there are data available for more than the 14,541 pregnancies mentioned above. The number of new pregnancies not in the initial sample (known as Phase I enrollment) that are currently represented on the built files and reflecting enrolment status at the age of 24 is 913 (recruited during Phases II, III, and IV, respectively). The phases of enrollment are described in more detail in the cohort profile paper and its update. The total sample size for analyses using any data collected after the age of 7 is therefore 15,454 pregnancies, resulting in 15,589 fetuses. Of these 14,901 were alive at 1 year of age.

The sampling strategy and attrition in ALSPAC has been extensively discussed. The cohort may be viewed as a normal-risk sample, for example, based on a 5% rate of preterm birth and 77% rate of small for gestational age. Key findings indicate that the low rate of minority participation was comparable to the Avon region, although lower than national figures; retention has been stronger for families with female children and for those at higher socioeconomic standing (Boyd et al., 2013). Accordingly, we include measures of child gender and socioeconomic standing in the current analyses. For the current study, our focus was assessments that included direct in-person assessments, which began for the entire cohort at approximately age 7 years. Because of our interest in examining individual differences in neurodevelopmental outcomes in typically developing children, we included live singleton births and excluded, on an a priori basis, those children who were born less than 2,500 g or before 37 weeks gestation. The current analyses focus on mothers on whom there were valid data on prenatal maternal infection and anxiety and child neurodevelopment outcomes in middle to late childhood (*n* = 7,042). Informed consent and assent were obtained from participants; ethical approval was obtained from the ALSPAC Ethics and Law Committee and from Local Research Ethics Committees (Bristol and Weston Health Authority: E1808 Children of the Nineties: Avon Longitudinal Study of Pregnancy and Childhood (ALSPAC); Southmead Heath Authority: 49/89 Children of the Nineties; Frenchay Health Authority: 90/8 Children of the Nineties; United Bristol Health care Trust: E4445 Alspac Focus at Eight).

### Measures

#### Prenatal Exposures

Prenatal infection covering the period from late first through second trimester was based on maternal endorsement of several conditions from a questionnaire administered at approximately 32 weeks gestation. Mothers reported whether or not they had the following conditions in the past 3 months: nausea, vomiting, diarrhea, vaginal bleeding, jaundice, urinary infection, cold, influenza, rubella, thrush, genital herpes, or “other infection.” We subsequently classified these conditions, on an a priori basis, according to the likelihood of active systemic infection. Conditions associated with immune activation or infection were considered part of a composite measure of prenatal infection: diarrhea, urinary infection, influenza/flu, thrush/candida, and herpes (rubella, which was also queried, was endorsed by just one of 7,042 respondents). We calculated a sum of reported infections and a dichotomous index of presence/absence of any infection. For sensitivity analyses, we also computed a broader measure of infection that included other self-reported exposures (including vaginal bleeding and “other infection”), a measure that included only self-reported influenza, and self-reported use of medicine for “infection.”

Maternal prenatal anxiety was based on the anxiety subscale of the Crown-Crisp experiential index (CCEI), a well-validated inventory that has been used in previous analyses of prenatal distress and child outcomes (Birtchnell et al., 1988; O'Connor et al., 2002). In addition, prenatal depressive symptoms were based on the Edinburgh postnatal depression scale (EPDS), a well-validated 10-item questionnaire (Cox et al., 1987). Both measures were assessed at approximately 32 weeks gestation; we also consider these measures at 8 weeks postnatal as a control for postnatal symptoms. Internal consistencies for all measures (based on alpha) were > .80.

#### Child Neurodevelopment and Psychiatric Symptoms

Several measures were collected to assess neurodevelopment and psychiatric symptoms when the children were approximately 8 years of age. The full-scale intelligence quotient (IQ) from the Wechsler Intelligence Scale for Children (WISC III, 3rd U.K. edition; Weschler et al., 1992) was administered at in-person assessment that took place at a half-day clinic. Except for the coding subtest, which was administered in the standard form, administration of the WISC involved alternate questions for every subtest, starting with the first item, to shorten the test. Social and communication problems associated with autism were assessed using the Social and Communication Disorders Checklist (SCDC; Skuse et al., 2005), a 12-item maternal report screening measures that assesses autistic-like traits, including problems in social reciprocity and verbal/nonverbal communication (sample items include, “Not aware of other people’s feelings”; “Does not pick up on body language”); the measure has been used widely in other research and has demonstrated high reliability and validity (St Pourcain et al., 2018); internal consistency (alpha) was .88. Higher SCDC scores reflect more social-communication deficits.

Symptoms of ADHD, anxiety, depression, and disruptive behavior were derived from the Development and Well-Being Assessment (DAWBA; Goodman et al., 2000), a validated psychiatric assessment tool widely used in epidemiological studies (Ford et al., 2003). The parent-completed DAWBA questionnaire constituted the primary source of information for the diagnosis of psychiatric disorders. In addition, where available, complementary sources of information on a child's emotional, behavioral, and academic characteristics were consulted, which included teacher version of the DAWBA that addressed potential hyperactivity and conduct disorder, teacher-reported Strengths and Difficulties Questionnaire (SDQ), and test results from reading and spelling competency. Number of symptoms for each disorder was recorded and diagnostic assessments were coded according to *DSM–IV* criteria by two experienced psychiatrists taking into account symptoms and burden or impact.

#### Covariates and Potential Effect Modifiers

Maternal education and household crowding at time of pregnancy were used as measures of socioeconomic status. Women endorsed their highest educational achievement within the U.K. educational system according to four categories (1 = *Certificate of Secondary Education/vocational training;* 2 = *O-levels, equivalent to modern day General Certificate of Secondary Education*; 3 = *A-levels, comparable to college entrance examinations*; 4 = *university degree or higher degree*). Household crowding was calculated by dividing the number of people in the household by the number of rooms; four categories were created ranging from low to high (1 = *0–.50*; 2 = *.50–.75*; 3 = *.75–1.00*; 4 = *> 1.00*). Mothers reported whether or not they were ever diagnosed with a severe mental illness (yes/no). Respondents also provided information on medication for prenatal depression and anxiety. Prenatal and perinatal covariates include parity, prenatal smoking (defined as the number of cigarettes smoked in the 2 weeks prior to assessment) and prenatal alcohol use (defined as the number of alcohol units in the week prior to assessment); prepregnancy BMI based on self-reported weight and height; gestational age (in weeks) and birth weight (in grams) extracted from medical records; maternal age in years was recorded at the completion of the initial prenatal questionnaire. Gestational age at the completion of the prenatal questionnaire, child sex, and child age (in months) at the time of testing were also considered as possible covariates.

### Data Analysis

The main analyses, which appear after descriptive data, were based on a definition of prenatal infection from the composite measure of symptoms indicating active systemic infection (diarrhea, urinary infection, influenza/flu, thrush/candida, and herpes); number of infections were categorized as 0, 1, 2, and 3 or more. Child outcomes were cognitive ability based on in-person IQ testing; social and communication problems associated with autism from parent report; and number of psychiatric symptoms from the diagnostic interview. Hypothesis testing was based on generalized linear models (McCullagh & Nelder, 1998), which have several advantages over ordinary linear regression; for example, error distributions are not considered normal, response variables are not required to have normal distributions, parameters are assessed according to maximum likelihood estimation. B coefficients using the existing scaling of the predictor variables were first estimated; in order to obtain beta coefficients, the models were reanalyzed using standardized variables. Generalized linear models for hypotheses were conducted in SPSS (Version 26). Based on the existing literature and available data, we included the following covariates on an a priori basis in all prediction models: maternal age; prepregnancy BMI because it has been considered as an index of maternal prenatal inflammation; birth weight and gestational age because they are measures of perinatal risk that may be associated with prenatal exposures; prenatal smoking because it is an additional index of prenatal risk exposure that may be confounded with target prenatal exposures and child outcomes; maternal education and household crowding because they provide a measure of socioeconomic status (and household crowding may also index prenatal illness exposure); postnatal maternal anxiety was included to test the specific prediction from prenatal anxiety; child age at assessment was included to adjust for variation in age at assessment; child sex was included because it is associated with many neurodevelopmental outcomes. None of the other covariates listed above were retained in prediction models because none was reliably associated with child outcomes. Sensitivity analyses considered alternative scaling and measurement of prenatal infection. A series of exploratory analyses examined moderation of effects by child sex and moderation of effects between the two target prenatal exposures; additionally, although our focus is on prenatal anxiety, we also examined prenatal depression in supplementary analyses. Given the large sample size, we attended to effect sizes for interpretation.

## Results

Descriptive and demographic data on the sample are provided in Table 1 (*n* = 7,042 pregnancies). Comparisons between those with no versus those with any infection identify a number of differences. Using Cohen’s *d* as a measure of effect size ([*M*_2_-*M*_1_]/*SD*_pooled_), the most notable difference, .37, was for prenatal anxiety; smaller (<.15) but notable differences were detected for smoking and prepregnancy BMI. Of the 7,042 women in this study, 32.7% reported one infection in pregnancy, 8.2% reported two infections, and 1.4% reported three or more infections; that is, 42.3% reported any infection in pregnancy. Self-reported rates of all types of infection varied: 6% for influenza, 5% for urinary infection, 29% for diarrhea, 13% for thrush/candida, and .3% for herpes. The broader category of infection, which also included vaginal bleeding and nonspecified “other infection” identified 46%. Rates of child psychiatrist-confirmed disorders were low (2% for any ADHD disorder; 3.1% for any oppositional-conduct disorder; 2.9% with any anxiety disorder; .4% for any depressive disorder, and .4% for any pervasive developmental disorder). Accordingly, prediction analyses focus on number of psychiatric symptoms rather than the presence/absence of disorder.[Table tbl1]

Of the 7,042 participants identified for the analyses, the rate of missing data was <5% on covariates and predictors, with two exceptions: prepregnancy BMI had a missing data rate greater than 5% (6,506 cases were available from the 7,042) and in-person IQ data were available on 5,213/7,042. There were 6,800 children with data on the SCDC and 6,866 with data on the DAWBA.

### Preliminary and Bivariate Analyses

Bivariate correlations, displayed in Table 2, indicate modest overlap between prenatal infection, prenatal anxiety, smoking, and sociodemographic risk. There was a modest positive association between prenatal infection and prenatal anxiety, as shown in the correlation table and according to the means when assessed for 0–4 infections: means (*SD*) of prenatal anxiety according to number of infections were 4.32 (3.23) for zero infections, 5.41 (3.53) for one infection, 5.99 (3.70) for two infections, and 7.73 (4.20) for three or more infections; *F*(3, 6833) = 96.57, *p* < .001). A similar pattern was obtained for prenatal depression: the means (*SD*) of EPDS according to number of infections were 5.98 (4.65) for zero infections, 7.25 (4.88) for one infection, 8.08 (5.11) for two infections, and 9.98 (6.07) for three or more infections; *F*(3, 6980) = 71.01, *p* < .001). In addition, prenatal infection and prenatal anxiety were also modestly associated with prenatal smoking.[Table tbl2]

### Prenatal Prediction of Child IQ

Bivariate analyses indicated that child IQ, based on the full-scale WISC, showed a dose–response pattern of association with number of maternal prenatal infections, that is, each increase in infection was associated with lower IQ (means [*SD*] were 106.21 [16.21] for zero infections, 105.00 [16.52] for one infection, 104.40 [15.51] for two infections, and 100.11 [15.12] for three or more infections; *F*(3, 5209) = 5.46, *p* < .001); similar dose–response patterns were obtained for the Verbal IQ (means [*SD*] were 109.08 [16.73] for zero infections, 107.76 [16.64] for one infection, 106.96 [16.10] for two infections, and 102.60 [14.01] for three or more infections; *F*(3, 5239) = 6.24, *p* < .001) and for Performance IQ (means [*SD*] were 101.28 [16.76] for zero infections, 100.48 [17.24] for one infection, 100.22 [16.43] for two infections, and 97.06 [16.15] for three or more infections; *F*(3, 5228) = 2.23, *p* = .08). The bivariate association between full-scale IQ and prenatal maternal anxiety was small in magnitude [*r*(5213) = −.07, *p* < .001]. However, in the regression model that adjusted for covariates, neither prenatal infection nor prenatal anxiety was significantly associated with child IQ (Table S1 in the online supplementary materials).

### Prenatal Prediction of Social and Communication Deficits

**Figure 1 fig1:**
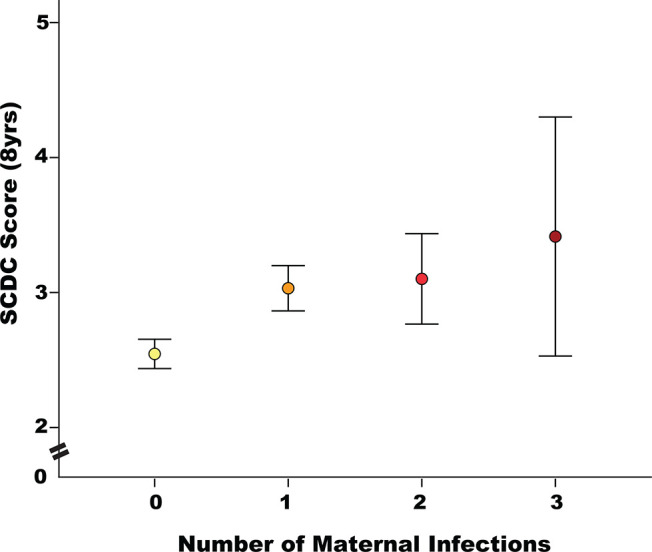
Means (SE) of SCDC Score at Age 8 Years According to Number of Prenatal Maternal Infections (0, 1, 2, 3+), Adjusting for Covariates *Note*. See the online article for the color version of this figure.

As shown in Figure 1, there was evidence of a dose–response pattern between prenatal infection and problem severity on the SCDC: mean problem severity on the SCDC increased with each prenatal infection reported (means [*SD*] were 2.53 [3.39] for zero infections; 3.01 [3.95] for one infection; 3.10 [3.98] for two infections; 3.41 [4.29] for three or more infections; *F*(3, 6796) = 11.37, *p* < .001). Bivariate correlation analysis indicated that problems on the SCDC were significantly and modestly associated with prenatal anxiety [*r*(6769) = .15, *p* < .001].[Fig fig1]

Regression analyses predicting continuous scores on the SCDC from prenatal exposures and covariates are presented in Table 3. Results indicate that both prenatal infection (scored 0 to 3 or more) and prenatal anxiety were both significantly associated with greater disturbance in social and communication problems after adjusting for covariates. The (adjusted) increase in score between none and 3 or more infections (i.e., .4) is small, but notable in this nonselected community sample.[Table tbl3]

Sensitivity analyses indicated that the prediction of SCDC was robust across alternative measures of prenatal infection. For example, the regression coefficient for none versus any infections was .30 (*SE* .10; *p* < .01). Similar effects, in terms of parameter estimates, were also found when specific infections were assessed separately, such as urinary tract infection (B .31 *SE* .21, *p* = .13) and influenza (B .42 *SE* .20, *p* = .04; the *SE* for these analyses were correspondingly higher given the smaller number of cases). Further sensitivity analyses indicated that including child full-scale IQ reduced, but only modestly, the effects of prenatal infection or prenatal anxiety on SCDC (for prenatal anxiety, the likelihood ratio chi-square was 16.99, *p* < .001 vs. 11.58, *p* < .001 for the model that included IQ as a covariate; for prenatal infection, the likelihood ratio chi-square was 9.89, *p* = .019 vs. 7.51, *p* = .057 for the model that included IQ as a covariate). Additionally, although there was a sizable main effect of sex on SCDC, there was not significant evidence that the prediction of SCDC differed significantly by sex for either prenatal exposure (*p* = .278 for the interaction between child sex and prenatal infection; *p* = .182 for the interaction between child sex and prenatal anxiety). There was also no evidence of a significant interaction between prenatal infection and prenatal anxiety in predicting SCDC: the interaction term was nonsignificant in the model (*p* = .537); Figure S1 in the online supplementary material displays the additive effects of prenatal infection and prenatal anxiety on SCDC. Lastly, analyses that included prenatal depression rather than anxiety indicated substantively similar effects (see Table S2 in the online supplementary materials).

### Prenatal Prediction of Psychiatric Symptoms

Regression analyses for psychiatric symptoms based on the DAWBA are reported in Table 4. Results for symptoms of ADHD was strikingly similar to those for SCDC. That is, both prenatal infection and prenatal anxiety independently predicted ADHD symptoms after adjusting for covariates. Effect size estimates (adjusting for all covariates) indicated a difference of 1.35 symptoms of ADHD between those exposed to none versus three or more infections. A series of supplementary analyses, parallel to those for SCDC (above), confirmed the robustness of these results. For example, supplementary analyses indicated that the prediction from prenatal infection to ADHD symptoms was robust to alternative scaling of prenatal infection (e.g., the prediction from any infection was B = .73 [*SE* .20], *p* < .001; for influenza only, B = 1.29 [*SE* .37], *p* = .001). Furthermore, there was no evidence of a significant difference in prediction from either prenatal exposure by child sex (*p* = .275 for the interaction between prenatal infection and child sex; *p* = .624 for the interaction between prenatal anxiety and child sex); neither was there evidence of a significant interaction between prenatal anxiety and prenatal infection in predicting ADHD symptoms (*p* = .856 for the interaction).[Table tbl4]

Results from the same prediction model (see Table 4) indicate that prenatal maternal anxiety was a significant predictor of child anxiety and depressive symptoms, adjusting for covariates; the prediction of disruptive behavior was weaker and not significant at *p* < .05. In contrast, prenatal maternal infection was reliably (*p* < .05) associated with child anxiety symptoms but only marginally with depressive and disruptive behavior symptoms. The effect size of prenatal prediction, in terms of the impact on numbers of symptoms, varied considerably. In the case of prenatal infections, the difference in child symptoms between those whose mothers reported no infections and those whose mothers reported three or more was 1.35 for ADHD but .29 for anxiety, and −.06 for disruptive behavior.

## Discussion

Analyses of this prospective longitudinal study of a large community sample indicated that prenatal anxiety and infection were reliably, independently, and additively associated with ADHD symptoms and social and communication difficulties, two key markers of neurodevelopment in middle childhood. The robustness of the prediction is implied from a dose–response pattern across an 8-year period from exposure to outcome, detailed clinical assessment, consistency across multiple definitions of prenatal infection, large sample size, and adjustment for perinatal and postnatal confounds. An additional novel and notable finding is that the prediction from prenatal infection extended to child anxiety (and was marginal for depressive and disruptive behavior symptoms). The findings further substantiate a developmental and conceptual model of psychopathology in which clinical disturbances can be traced to prenatal exposures, and raise novel questions about mechanisms of action, and their timing of influence.

Research findings suggest that multiple types of prenatal exposure, from chemical and pollution agents and specific micronutrients to, as in the current study, prenatal infection and mood disturbance, are reliably associated with child health and development. A key conceptual point in these studies, and the DOHaD and MIA models with which they are affiliated, is that brain and behavioral development begin before birth and are susceptible to prenatal exposures. However, a key limitation of these studies is the focus on a singular prenatal exposure, which may mis-specify effects and implied mechanisms to the extent that different types of risk exposures are confounded—as is often found. Furthermore, many specific types of risks are inherently variegated. For example, studies of the Dutch Hunger Winter (Schulz, 2010) focus on nutritional deprivation, but the impact of the exposure likely also derives from prenatal distress and changes in many kinds of health behaviors, exposures, and health care. Similarly, studies of pregnant women during the period affected by the severe acute respiratory syndrome coronavirus 2 (SARS-CoV-2) pandemic must account for increases in stress as well as the multiple kinds of changes in economic, nutritional, health care, and health behaviors that may affect the health of the mother, pregnancy, and child—rather than just one or other of these. To that end, we sought in the current article to examine two of the more prevalent and robust risk factors for child neurodevelopment that derive from separate research traditions, with disjunctive conceptual models and wholly separate empirical bases.

Much is known about how the health of the fetus may be disrupted by prenatal infection by viruses, bacteria, and parasites (Adams Waldorf & McAdams, 2013). Importantly, there is growing recognition that prenatal infections that may shape fetal brain development extend beyond the classic TORCH agents (*Toxoplasma gondii*, rubella, cytomegalovirus, herpes simplex virus) to a much broader array of agents associated with prenatal inflammation, which may be driven by psychological symptoms, stress, or health behaviors such as obesity. Links between inflammation and developmental and physical disability (Grether & Nelson, 1997) and brain disorders (Brown et al., 2005; Croen et al., 2019) have been noted for some time, but there has been limited attention to competing exposures and very few studies consider the broader phenotypes of neurodevelopment and mental health. In that context, the current study is unique in assessing a broad spectrum of neurodevelopmental and psychiatric conditions in young children from parent and clinician evaluation, variability in symptom expression (rather than disorder presence/absence), and consideration of covariation between prenatal infection and prenatal anxiety.

The current findings extend the MIA model by demonstrating dose–response patterns of outcomes across a continuous range of symptomatology. Specifically, maternal prenatal infection was associated with a continuous trait of social and communication problems assessed in a large community sample. The implication is that prenatal infection may have an even larger public and clinical health impact because of its influence on the broader (endo)phenotype underlying a clinical diagnosis of autism. A more novel prediction was to child anxiety symptoms. That is significant insofar as it implies that the psychiatric and behavioral phenotypes—and their underlying neurodevelopmental mechanisms—that may originate with prenatal infection may be broader than autism and schizophrenia, which have dominated this line of clinical research.

The findings suggest some specificity in effects. So, for example, the largely independent predictions from prenatal infection and anxiety imply distinct and specific underlying mechanisms: additive, independent prediction would not result if these exposures operate via wholly shared mechanisms. Our findings do not directly implicate inflammatory or stress physiology mechanisms, but they do indicate that prenatal infection and prenatal anxiety operate separately. That is a novel observation that requires detailed studies of maternal prenatal biology. Second, the predictions from prenatal infection were slightly less robust than those from prenatal anxiety, after adjusting for covariates (see Table 4). We also found a lack of significant prediction to disruptive behavior from prenatal infection, after adjusting for covariates; notably, there was also a weaker and nonsignificant prediction of disruptive behavior from prenatal anxiety (e.g., compared to other symptom clusters). Individual differences in disruptive behavior may simply be less reliably predicted from these prenatal exposures, or it may be that the prenatal prediction from these factors is confounded by covariates and postnatal exposures. Understanding why there may be differential prediction from prenatal exposures to diverse symptom clusters, from social communication disorders to disruptive behavior symptoms, may shed light on brain mechanisms and preventive intervention strategies. Two further findings are notable: (a) the association between prenatal infection and both social behavior and communication and attention problems was not explained by general cognitive ability, and (b) the prediction of child outcomes was from infection and not from prepregnancy BMI, a broadly inflammatory state that had been associated with neurodevelopment in prior studies; prepregnancy BMI was not a reliable predictor of any of the symptom clusters.

Further research is now needed to contrast alternative mechanisms that may underlie prenatal anxiety and infection in order to provide clinical guidance. Research on how prenatal immune mechanisms may alter child health outcomes is particularly needed; there remain basic questions in this area of study even in preclinical research (Careaga et al., 2017). An immune-focused model derives partly from the basic task of pregnancy in which the mother must make immune adaptations to accommodate the semiallograft—the fetus. Disruptions in this adaptation to the fetus may underlie major histopathological markers of inflammation in the placenta, such as chorioamnionitis and chronic villitis (Kim et al., 2015), which have be linked with brain morphology in animal models (Burd et al., 2011) and, in children, neurodevelopment (Chau et al., 2014). A prevailing model was that pregnancy was an immunosuppressive state and that a successful pregnancy was dependent on a shift to a Th2/anti-inflammatory response, although research has considered a wide array of pro- and anti-inflammatory cytokines such as interleukin(IL)-6, IL-17 or TNF-alpha (Estes & McAllister, 2016). Research targeting specific inflammatory markers in pregnancy in relation to child brain and neurocognitive outcomes is attracting intense interest (Rasmussen et al., 2022), and will shape the next phase of this line of study. Even if inflammatory mechanisms are involved, they may act via several pathways, including glucocorticoid signaling cascades or metabolic markers such as leptin. Also, a focus on the innate immune system may be too limited. Findings linking T cell function to early neurodevelopment (Filiano et al., 2015) and recent findings on complement 4 genes and schizophrenia (Sekar et al., 2016) offer two disparate but potentially equally compelling examples of how the study of immune mechanisms and early brain development will require the integration of a broader rather than singular approach to measuring the immune system.

Evidence of sex moderation of prenatal exposures is inconsistently reported and may be dependent on outcome phenotype and age of assessment. For example, analyses from the Collaborative Perinatal Project suggested that the association between severe infection and neurocognitive outcomes may be greater in males (Lee, Papandonatos, et al., 2020); data from the New England family study suggested that prenatal immune programming for depression in adults may be stronger for females (Gilman et al., 2016). Child sex had the expected sizable main effect on child neurodevelopmental outcomes in the current study, but did not moderate the association between prenatal infection or anxiety and child outcomes—despite the large sample size for detecting moderation (> 3,000 males and > 3,000 females). Further mechanistic research is needed to account for the reported sex moderation effects of prenatal exposures and why, as implied in this and other studies, there is inconsistency across study and exposure.

There are several limitations of the current study. The first is limit of longitudinal cohort studies for deriving causal effects. We conducted prospective longitudinal analyses and statistically adjusted for confounders but, like other human studies in this area, we are limited in drawing causal connections. A further limit is that we did not have adequate leverage to discern specific timing effects. There is a sizable literature suggesting that early to midgestation (which was largely covered in the current assessment) is a vulnerable period in fetal brain development (Sham et al., 1992), although there are conflicting findings (Li et al., 2009). In this regard, it is notable that other analyses failed to find substantial effects of timing of the prenatal infection data on mental health (Zammit et al., 2009). Also, we were reliant on self-reports of infection and, like virtually all studies in this area, we did not have direct placental measures, which are needed to develop a mechanistic model (O'Connor et al., 2019). Finally, questions remain about the nature of affective disturbance in the perinatal period and how it is best measured (Cochran et al., 2020). The current focus on anxiety follows a sound conceptual and empirical base, and we did find that the associations were comparable for depressive symptoms—suggesting an effect that is robust across different measures of maternal prenatal distress. Importantly, these limitations are offset by several strengths of the study, including a prospective longitudinal design and analysis of nearly a decade of data; assessment of child neurodevelopment using questionnaire and in-person assessment; extensive data on prenatal exposures and covariates; consideration of a broad set of infections and illnesses; a large community sample.

Several clinical implications of the findings deserve attention. Perhaps the strongest is the need for additional clinical studies to support prenatal mental health because this may benefit both the mother and child. There are likely benefits to the mother and child of promoting prenatal immune health and reducing inflammation, in response to common systemic infections or infectious diseases, or other exposure types. If, and how, prenatal interventions to change health behavior may promote maternal psychological and physical health and the developing child is an active area of study for example, (Dodd et al., 2016). Vaccination is another intervention needing attention in this regard. The benefits of common prenatal vaccinations continue to show strong safety records for child health; this matter continues to attract extensive clinical research attention (e.g., Mehrabadi et al., 2021), and this has been further prompted most dramatically by the SARS-CoV-2 pandemic. Reducing prenatal systemic infection and psychological distress are important intervention strategies for promoting child neurodevelopment and mental health. Further work of this kind may reveal valuable and applicable assessment strategies (e.g., concerning inflammatory markers), and may offer adjunctive treatments options. Intervention work of this kind is also needed to complement the growing interest in applying neuroinflammation-related hypotheses to child neurodevelopmental and behavioral health outcomes (O’Connor et al., 2014; Oddy et al., 2018).

## Supplementary Material

10.1037/abn0000746.supp

## Figures and Tables

**Table 1 tbl1:** Cohort Descriptive Data

Maternal and perinatal characteristics	Total	No infection	Any infection	*F*/chi-square (*df*)
Maternal age	28.66	28.81	28.45	11.04 (1,6,914)
	(4.54)	(4.48)	(4.61)	*p* = .001
Education	3.21	3.23	3.17	3.55 (1,7,016)
	(1.23)	(1.23)	(1.23)	*p* = .060
Prepregnancy BMI	22.85	22.69	23.08	18.84 (1,6,504)
	(3.63)	(3.47)	(3.83)	*p* < .001
Crowding	1.77	1.73	1.83	25.80 (1,6,844)
	(.87)	(.85)	(.89)	*p* < .001
Prenatal smoking	1.22	.97	1.55	36.54 (1,6,972)
	(3.92)	(3.45)	(4.51)	*p* < .000
Birth weight (g)	3,510.13	3,506.41	3,515.21	.67 (1,7,040)
	(444.15)	(448.72)	(437.85)	*p* = .412
Gestational age (wks)	39.79	39.78	39.81	.70 (1,7,040)
	(1.29)	(1.31)	(1.26)	*p* = .404
Prenatal anxiety	4.85	4.32	5.59	153.42 (1,6,839)
	(3.45)	(3.23)	(3.62)	*p* < .001
Child characteristics				
Sex (% male]	51% (3,595)	51% (2,060)	52% (1,535)	.47 (1)
				*p* = .499
IQ (full**-**scale)	105.59	106.21	104.73	10.48 (1,5,211)
	(16.26)	(16.21)	(16.30)	*p* = .001
Symptoms				
SCDC	2.75	2.53	3.04	32.82 (1,6,798)
	(3.65)	(3.39)	(3.96)	*p* < .001
ADHD	4.81	4.39	5.39	36.13 (1,6,762)
	(6.78)	(6.38)	(7.25)	*p* < .001
Depressive	.31	.27	.36	11.72 (1,6,887)
	(1.01)	(.95)	(1.09)	*p* = .001
Disruptive behavior	.19	.16	.24	13.16 (1,7,002)
	(.95)	(.86)	(1.07)	*p* < .001
*Note*. Highest educational achievement was scored using UK educational system according to four categories (1 = *Certificate of Secondary Education/vocational training*; 2 = *O-levels, equivalent to modern day General Certificate of Secondary Education*; 3 = *A-levels, comparable to college entrance examinations*; 4 = *university degree or higher degree*); household crowding was calculated by dividing the number of people in the household by the number of rooms (1 = *0–.50*; 2 = *.50–.75*; 3 = *.75–1.00*; 4 = *> 1.00*).												

**Table 2 tbl2:** Associations Between Pre- and Perinatal Risks

Variable	1. Prenatal infection	2. Prenatal anxiety	3. Maternal age	4. Birth weight	5. Gestational age	6. Prenatal smoking	7. Prepregnancy BMI	8. Maternal education	9. Crowding	10. Child sex	11.IQ	12.SCDC	13.ADHD symptoms	14. Anxiety symptoms	15. Depressive symptoms
1															
2	.18														
3	−.04	−.09													
4	.01	.00	.07												
5	.01	−.02	−.06	.32											
6	.07	.12	−.10	−.13	.00										
7	.05	.01	.00	.18	.01	.01									
8	−.02	−.10	.26	.03	.00	−.21	−.12								
9	.06	.12	−.13	.04	.00	.17	.06	−.26							
10	−.01	.02	−.04	−.12	.04	.01	−.01	.01	.00						
11	−.05	−.07	.17	.07	−.01	−.09	−.08	.37	−.20	.00					
12	.07	.15	−.04	−.02	.00	.07	−.02	−.05	.04	−.12	−.13				
13	.07	.16	−.07	−.04	−.01	.08	−.01	−.07	.06	−.16	−.21	.67			
14	.05	.17	.01	−.03	.02	.02	−.05	.08	−.03	.00	.00	.26	.26		
15	.04	.14	−.03	−.02	.00	.04	.01	.01	.02	.02	−.03	.21	.22	.38	
16	.04	.09	−.02	−.01	.01	.08	.01	−.03	.04	−.07	−.06	.53	.44	.21	.19
*Note*. Prenatal infection in this table is measured dichotomously (1 = infection reported, 0 = no infection). Correlations greater than .03 are significant at *p* < .01.															

**Table 3 tbl3:** Prenatal Prediction of Child Social and Communication Disorders Checklist

			Likelihood ratio	
Predictor	β	B (*SE*)	Chi-square (*df*)	*p*
Prepregnancy BMI	−.06	−.02 (.01)	1.40 (1)	.236
Child sex (female)	−.90	−.90 (.09)	94.23 (1)	.000
Prenatal anxiety	.24	.07 (.02)	16.99 (1)	.000
Postnatal anxiety	.44	.14 (.02)	58.81 (1)	.000
Prenatal infection			9.89 (3)	.019
0 infections		—		
1 infection	.30	.30 (.10)		
2 infections	.28	.28 (.17)		
3+ infections	.40	.40 (.43)		
Birth weight	−.09	.00 (.001)	2.98 (1)	.084
Gestational age	.08	.06 (.04)	2.65 (1)	.104
Maternal age	−.04	−.01 (.01)	.66 (1)	.417
Child age at assessment	.02	.01 (.03)	.22 (1)	.640
Maternal education	−.10	−.09 (.04)	4.16 (1)	.041
Crowding	.01	.01 (.06)	.02 (1)	.885
Prenatal smoking	.16	.04 (.01)	10.21 (1)	.001
*Note*. β coefficients, provided for effect size comparisons, were based on rerunning the generalized linear model with standardized predictor variables.					

**Table 4 tbl4:** Prenatal Prediction of Psychiatric Symptoms

	1. ADHD			2. Anxiety			3. Depressive			4. Disruptive behavior		
			Likelihood ratio			Likelihood ratio			Likelihood ratio			Likelihood ratio
			Chi-square			Chi-square			Chi-square			Chi-square
Predictor	b	B (*SE*)	(*df*), *p*	b	B (*SE*)	(*df*), *p*	b	B (*SE*)	(*df*), *p*	b	B (*SE*)	(*df*), *p*
Prepregnancy BMI	−.02	−.01 (.02)	.043 (1)	−.03	−.01 (.01)	2.39 (1)	.03	.01(.004)	3.31 (1)	.004	.00 (.04)	.11 (1)
			*p* = .836			*p* = .122			*p* = .069			*p* = .742
												
Child sex (female)	−2.25	−2.25 (.17)	169.63 (1)	−.03	−.03 (.04)	.47 (1)	.04	.04 (.05)	2.21 (1)	−.14	−.14 (.02)	32.41 (1)
			*p* < .000			*p* = .491			*p* = .137			*p* < .000
Prenatal anxiety	.44	.13 (.03)	16.85 (1)	.15	.04 (.01)	28.51 (1)	.08	.02 (.01)	25.14 (1)	.03	.01 (.01)	2.75 (1)
			*p* < .000			*p* < .000			*p* < .000			*p* = .097
Postnatal anxiety	.93	.29 (.03)	76.20 (1)	.26	.08 (.01)	89.89 (1)	.09	.03 (.01)	32.90 (1)	.09	.03 (.01)	30.05 (1)
			*p* < .000			*p* < .001			*p* < .000			*p* < .000
Prenatal			9.14 (3)			9.83 (3)			6.56 (3)			7.31 (3)
infections			*p* = .027			*p* = .020			*p* = .087			*p* = .063
0		—			—			—			—	
1	.49	.49 (.19)		.00	.00 (.05)		.01	.01 (.03)		.07	.07 (.05)	
2	.40	.40 (.32)		.23	.23 (.08)		.11	.11 (.06)		.07	.07 (.05)	
3+	1.35	1.35 (.80)		.29	.29 (.20)		.19	.19 (.12)		−.06	−.06 (.11)	
BW	−.40	−.001 (.0,002)	18.13 (1)	−.04	.00 (.00)	3.15 (1)	−.02	.00 (.00)	2.15 (1)	−.01	.00 (.00)	.89 (1)
			*p* < .001			*p* = .076			*p* = .143			*p* = .346
GA	.10	.08 (.07)	1.31 (1)	.05	.04 (.02)	5.37 (1)	.01	.01 (.01)	.52 (1)	.02	.02 (.02)	2.62 (1)
			*p* = .252			*p* = .021			*p* = .472			*p* = .107
Mat age	−.24	−.05 (.02)	6.71 (1)	.01	.00 (.00)	.06 (1)	−.02	.00 (.00)	1.26 (1)	−.01	.00 (.00)	.33 (1)
			*p* = .01			*p* = .813			*p* = .263			*p* = .567
Child age	.22	.14 (.05)	6.28 (1)	.05	.03 (.01)	4.71 (1)	.01	.01 (.01)	.90 (1)	−.01	.00 (.00)	.15 (1)
			*p* = .012			*p* = .030			*p* = .371			*p* = .694
Mat educ	−.21	−.17 (.08)	4.89 (1)	.14	.12 (.02)	36.42 (1)	.04	.03 (.01)	8.23 (1)	.00	.00 (.01)	.06 (1)
			*p* = .029			*p* < .001			*p* = .004			*p* = .805
Crowding	.13	.15 (.11)	2.01 (1)	−.05	−.06 (.03)	4.46 (1)	.01	.01 (.02)	.36 (1)	.02	.02 (.02)	1.75 (1)
			*p* = .156			*p* = .035			*p* = .549			*p* = .186
Prenatal smoking	.32	.08 (.02)	11.64 (1)	.03	.01 (.01)	2.29 (1)	.03	.01 (.00)	4.21 (1)	.08	.02 (.003)	31.38 (1)
			*p* < .001			*p* = .122			*p* = .040			*p* < .001
*Note*. BW = birth weight; GA = gestational age; Mat educ = maternal education. β coefficients, provided for effect size comparisons, were based on rerunning the generalized linear model with standardized predictor variables.
