# Supplementary material for: Maternal Prenatal Infection and Anxiety Predict Neurodevelopmental Outcomes in Middle Childhood
Source: J Psychopathol Clin Sci. 2022 Mar 3;131(4):422–34. doi: 10.1037/abn0000746 (PMC9069845; doi:10.1037/abn0000746)
Supplement: Supplementary file 1 [file ABN-2020-1947_Suppl1.docx]

Table S1. Prenatal prediction of Full Scale IQ

Likelihood ratio

B (SE) chi-square(df)

Pre-pregnancy BMI -.27 (.07) 17.04(1)***

Child sex (female) .54 (.46) 1.39(1)

Prenatal anxiety -.12 (.08) 2.09(1)

Postnatal anxiety -.02 (.09) .03(1)

Prenatal infection 3.90(3)

0 infections --

1 infection -.51 (.50)

2 infections -.49 (.84)

3+ infections -3.78 (2.15)

Birth weight .002 (.001) 19.69(1)***

Gestational age -.19 (.19) 1.05(1)

Maternal Age .27 (.05) 26.25(1)***

Child Age at Assessment -.38 (.15) 6.68(1)**

Maternal education 4.09 (.21) 377.17(1)***

Crowding -2.40 (.28) 71.04(1)***

Prenatal smoking .01 (.07) .02(1)

___________________________________________________________________________

Note. * p<.05, ** p<.01, *** p<.001.

Table S2. Prenatal depression prediction of Child Social and Communication Disorders Checklist

Likelihood ratio

B (SE) chi-square

Pre-pregnancy BMI -.02 (.01) 2.05(1)

Child sex (female) -.88 (.09) 88.05(1)***

Prenatal depression .05 (.01) 19.14(1)***

Postnatal depression .10 (.01) 61.39(1)***

Prenatal infection 12.39(3)**

0 infections --

1 infection .33 (.10)

2 infections .30 (.17)

3+ infections .50 (.42)

Birth weight .000 (.000) 3.53(1)

Gestational age .07 (.04) 3.33(1)

Maternal Age -.01 (.01) 1.45(1)

Child Age at Assessment .01 (.03) .15(1)

Maternal education -.06 (.04) 2.32(1)

Crowding -.01 (.06) .01(1)

Prenatal smoking .04 (.01) 11.30(1)***

___________________________________________________________________________

Note. * p<.05, ** p<.01, *** p<.001.

Figure S1. legend

Supplementary Figure. Additive effects of prenatal infection and prenatal anxiety on SCDC. Figure shows the mean SCDC scores in children whose mother had neither anxiety nor infection (-Anx/-Infection), not anxiety but infection (-Anx/+Infection), anxiety but not infection (+Anx/-Infection), both anxiety and infection (+Anx/+Infection).
