# Supplementary figures and images for: Maternal Prenatal Infection and Anxiety Predict Neurodevelopmental Outcomes in Middle Childhood
Source: J Psychopathol Clin Sci. 2022 Mar 3;131(4):422–34. doi: 10.1037/abn0000746 (PMC9069845; doi:10.1037/abn0000746)

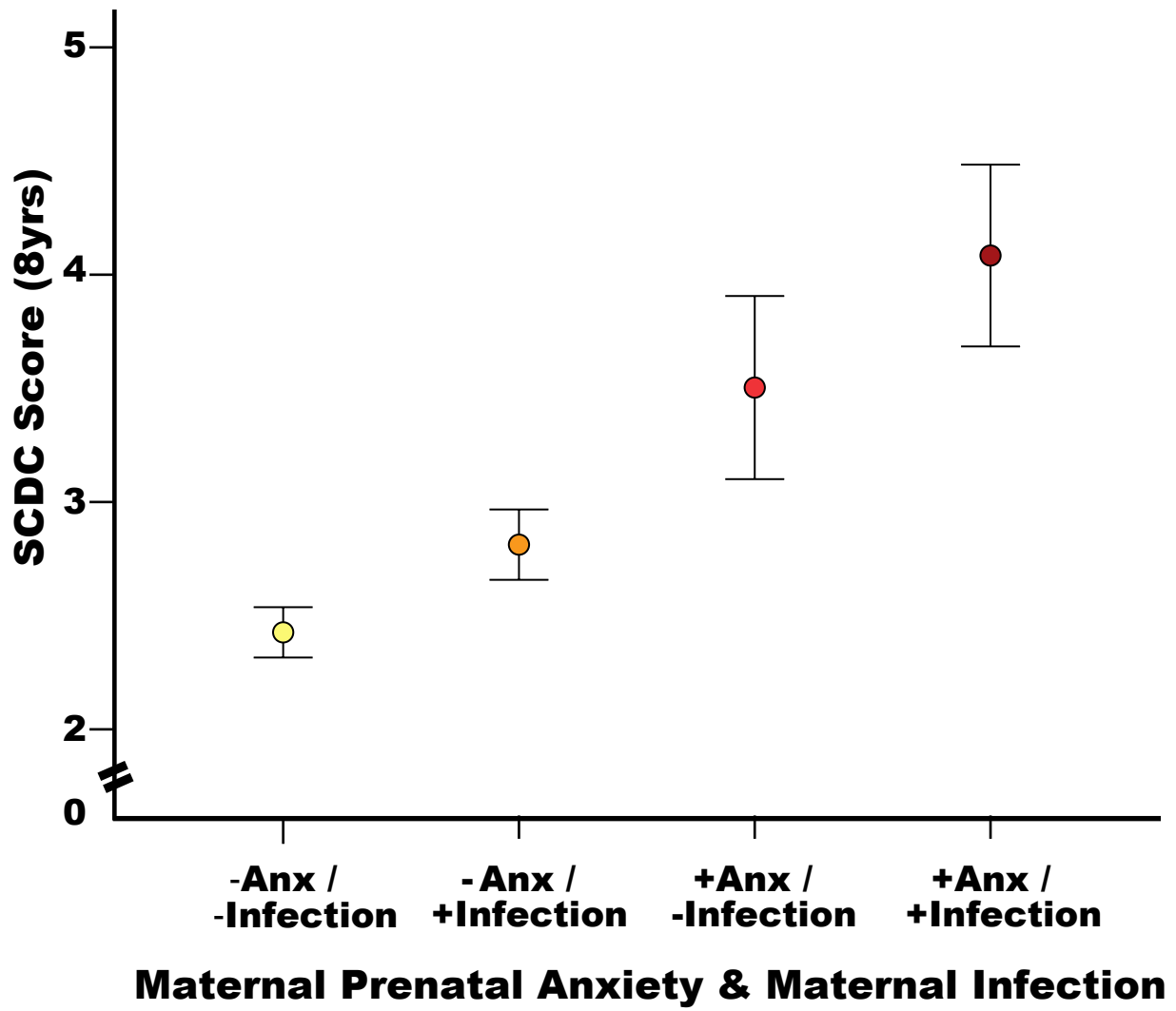

Supplement: Supplementary file 2 [file ABN-2020-1947_Suppl2.pdf]
